# Antioxidants and management of polycystic ovary syndrome in Iran: A systematic review of clinical trials

**Published:** 2015-01

**Authors:** Leila Amini, Najmeh Tehranian, Mansoureh Movahedin, Fahimeh Ramezani Tehrani, Saeedeh Ziaee

**Affiliations:** 1 *Department of Midwifery & Reproductive Health, Medical Sciences Faculty, Tarbiat Modares University, Tehran, Iran.*; 2 *Department of Anatomical Sciences, Medical Sciences Faculty, Tarbiat Modares University, Tehran, Iran. *; 3 *Reproductive Endocrinology Research Center, Research Institute for Endocrine Sciences, Shahid Beheshti University of Medical Sciences, Tehran, Iran.*

**Keywords:** *Polycystic ovary syndrome*, *Iran*, *Vitamin*, *Antioxidant*

## Abstract

**Background:**

Recently there is a focus on the antioxidants as adjuvant treatment of polycystic ovary syndrome (PCOS), the most endocrinopathy in reproductive age women.

**Objective:**

The aim of this review is answer to the question whether antioxidants are effective for managing of hormonal and metabolic problems in women with PCOS based on first degree evidences from Iran.

**Materials and Methods:**

A systematic review of clinical trials was done in Persian and international databases including PubMed, Scientific Information Database, Google Scholar, Iran Medex, and Magiran up to 2013. Keywords were including polycystic ovary syndrome, Iran, vitamin, antioxidant. From 440 potential studies found electronically, 11 studies; including 444 women in intervention and 390 women in control groups. Intervention in three studies was Calcium-vitamin D or calcitriol; in three studies was ω-3 fatty acids; in two studies was N-acetyl cysteine; in one study was folic acid; in one study was Zinc; and in one study was Soy.

**Results:**

Finally, 11 studies that were relevant and met the inclusion criteria reviewed. There were 7 studies in English and 4 studies in Persian. We couldn’t include all studies because all full texts were not accessible.

**Conclusion:**

The results showed that antioxidants and vitamins have positive effects on management of PCOS women. Although it seems more studies is necessary in this field.

## Introduction

Polycystic ovary syndrome (PCOS) is the most common endocrine disorder among reproductive-aged women with various prevalence from 5-21% (1, 2). This syndrome is associated with wide spectrum complications in different aspects of health, including reproductive (hyperandrogenism, hirsutism, anovulation, infertility, and menstrual disturbance), metabolic (obesity and diabetes mellitus as well as cardiovascular risk), and psychological features (mood disorders and decreased quality of life) (1, 3). 

Nevertheless, the management and treatment of this major problem is not deterministic, and surrounded by many controversies. Common PCOS managements only can control symptoms moderately and are not effective completely on prevention of complications. Recently, complementary and alternative medicine (CAM) has been discussed as an adjuvant medical management of PCOS. Several CAM treatments are studied and it seems they have some beneficial effects on the severity of PCOS and its endocrine, cardio metabolic, and reproductive complications (4). For instance, lifestyle modification, acupuncture, yoga, meditation, aromatherapy, homeopathy, Ayurveda, weight loss, herbal medicine, and antioxidants especially vitamins are considered as CAM in PCOS (5-13). 

Nowadays, the use of antioxidants in management of women with PCOS has attracted lots of interests. Some characteristics of PCOS such as obesity and abdominal adiposity, androgen excess, and insulin resistance can develop oxidative stress in these patients (14). Indeed, PCOS is a condition with significant decrease in serum antioxidant and vitamins levels and these women are in an increased risk of oxidative status (15). Oxidative stress and antioxidant decrease may lead these women to increased risk of cardiovascular disease, insulin resistance, hypertension, central obesity, and dyslipidemia (16-18).

Antioxidant supplementation has been shown to improve insulin sensitivity and other health threating conditions in women with PCOS (15, 16). Despite the important role of alternative medicine especially antioxidants in management of PCOS women, there are not many well-designed papers or detailed literature reviews report in this field, especially in Iran. In the other hand, the available studies addressing antioxidant use in PCOS women yielded controversial results, because of their sample sizes or the diversity in the prescribed antioxidant or outcomes assessed in them. For overcoming these limitations, updating our knowledge on this field and a critical appraisal of all available studies might be helpful to guide clinical practice. 

To date, there is no systematic review aimed to evaluate the efficacy of antioxidants in Iranian PCOS women especially based on systematically search and review on all available clinical trials in the literatures that were done in Iran. So, this systematic review conducted in order to answer the question whether antioxidants are effective for managing of hormonal and metabolic problems in women with PCOS.

## Materials and methods

**Definitions**

PCOS identified as a disorder of ovarian androgen excess. This syndrome is characterized by hyperandrogenism and/or hyperandrogenemia, oligoovulation, and exclusion of known disorders (Cushing’s syndrome, hyperprolactinemia, CAH, etc.). Although, the polycystic ovaries on ultrasound, is considered as forth criterion but there is no consensus about it (19, 20).

Management of PCOS: Management of PCOS is any ways to control of four main PCOS complications: irregular menses, hirsutism and infertility which are “acute” issues, and one important chronic issue or insulin resistance (21). Antioxidants: Antioxidants are substances that can protect the body from the highly reactive free radicals and oxygen species damage by converting the free radicals into more stable substances (22). Antioxidants can be generated endogenous (enzymatic), or received from foods or supplements (non-enzymatic) (23).

**Search strategy and engines**

We searched Literature on the antioxidants for treatment of PCOS that was acquired through searching the 3 Iranian database including Scientific Information Databases (SID), Iranian Biomedical Journal (Iran Medex), and Iranian Journal Database (Magiran) as well as international databases including PubMed/ Medline, and Google Scholar. The search was limited to the Persian and/or English language papers published until November 2013. For international databases, the search strategy was conducted depend on different combination of the terms "Polycystic Ovary Syndrome" (MeSH) OR PCOS AND “vitamin (MeSH) OR antioxidant (MeSH) AND Iran. All Iranian scientific databases were searched only using the keyword "Polycystic Ovary Syndrome" OR PCOS AND Iran.

**Inclusion and exclusion criteria**

The inclusion criteria were as follows: 1) studies in the mentioned databases with full text; 2) studies with experimental or quasi-experimental designs published in peer-reviewed journals; 3) studies with human samples (not animal studies). All studies about exercise were excluded. 

**Defining types of participants**

All of Iranian PCOS women in reproductive age who diagnosis of their PCOS was established according to Rotterdam criteria and had received a kind of antioxidants for PCOS complications management.

**Study selection and assessment of study quality**

For matching the inclusion criteria, all titles and abstracts of searched papers were critically assessed by two reviewers independently. This assessment was according to a checklist of aims, research question, and inclusion and exclusion criteria. Discords between reviewers were decided by consensus. After that, one reviewer assessed the quality of the included studies according to pre-defined criteria, including criteria of selection, blinding, randomization, methods of outcome assessment and data analysis. Studies were ranked based on having a degree of bias risk according to "the Cochrane Collaboration’s tool for assessing risk of bias" as follows: 1) Low risk of bias was assumed when there were few unfulfilled criteria with negligible effects on the study conclusions. 2) Moderate risk of bias was assumed when there were some unfulfilled criteria that may affect the study conclusions and raises some doubt about them, and 3) High risk of bias was assumed when there were few or no fulfilled criteria, and unfulfilled criteria were more likely to have serious effects on confidence of the study conclusions.

**Data extraction**

Data of all relevant studies were extracted by two reviewers independently. Then information was classified and summarized as follows: author, year of publication, language of paper, study design, study center, sample size (total and in each group), type and dosage of intervention, time and duration of intervention, and main outcomes. If the data was incomplete or more details were required, tried best to communicate with authors. 

**Article categorization**

**Figure 1:**
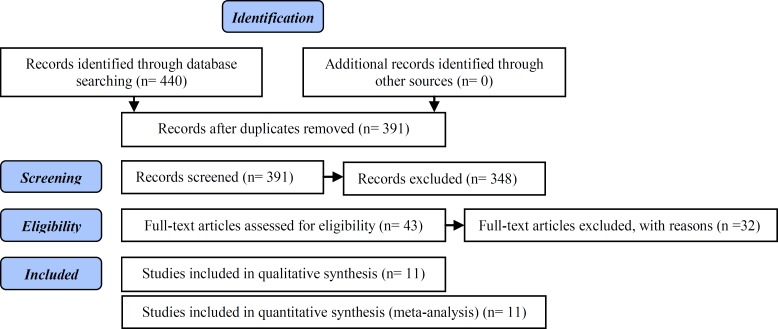
Article flow diagram

In this review, article categorization was performed as figure 1. 

## Results

According to the literature search, from 440 studies that were identified, just 43 studies were relevant and meet the inclusion criteria. From these studies, 29 studies were excluded because of duplication, and full text of 3 studies was not accessible. Finally, 11 studies; including 834 reproductive age women with PCOS (444 women in intervention and 390 women in control groups) were selected. There were 7 studies in English (24-30) and 4 studies in Persian (31-34). 

All of RCTs were included, even if the methodology was questionable. According to Costello *et al* including accurate questionable studies is necessary for having a complete perspective on the review (35). All samples of reviewed studies were women with PCOS. Table I shows more information about these studies.

**Table I:** Information about reviewed studies

**Author/ Language (ref)**	**Year**	**Place**	**Type of Study**	**Sample Size**	**Intervention**	**Int. Duration**	**Main Outcomes**
Khani et al English (24)	2011	Isfahan	Quasi-randomized trial	137	1-Soy (Genistein 18 mg /BD) 2-Placebo	3 months	**Reduce: **LH, TG, LDL, DHEAS, and T
Kazerooni et al English (25)	2008	Shiraz	Prospective clinical trial	70	Folic acid 1mg/d	3 months	**Reduce: **Homocysteine (HCY) in hyperhomocysteinemic PCOS women
Salehpour et al English (26)	2009	Tehran	Placebo-controlled double-blind randomized clinical trial	180	1-N-Acetyl Cysteine (NAC) 1.2 g/d +100 mg Clomiphene Citrate (CC) 2- CC+ placebo	5 days	**Increase: **Number of follicles greater than18 mm, Endometrial thickness on the day of hCG administration, Ovulation and pregnancy rates, No adverse side-effects and ovarian hyperstimulation syndrome
Salehpour et al English (27)	2012	Tehran	Prospective double-blind randomized clinical trial	46	1-N-Acetyl Cysteine (NAC) 1.8 g/d 2- placebo	6 weeks	**Increase: **Ovulation rate, HDL **Reduce: **Weight and BMI,Waist/hip ratio, FBS and Insulin, TC, LDL, HOMA-IR A
Rashidi et al English (28)	2009	Tehran	Randomized clinical trial	60	1- Calcium-D 2- Calcium-D+ metformin 1500 mg/d 3-metformin	3 months	**Increase (in group 2):** Number of dominant Follicles ≥ 14 mm, regular menses
Bonakdaran et al Persian (31)	2012	Mashhad	Randomized clinical trial	51	1-Metformin 1000 mg/day 2- Calcitriol 0.5 mic/d 3-Placebo	3 months	**Metformin improved: **Weight, BMI, Insulin, Insulin resistance. **Calcitriol improved: **PTH, Systolic Blood pressure, Ovulation
Mohammadi et al English (29)	2012	Kermanshah	Randomized clinical trial	44	1-omega -3 fatty acids 4 g/d 2-Placebo	3 months	**Increase: **Median and mean of follicular size
Rafraf et al Persian (33)	2012	Tabriz	Double-blind randomized controlled clinical trial	64	1-omega -3 fatty acids 4 g/d 2-Placebo	8 weeks	**Reduce: **TC/HDL-C and LDL-C/HDL-C ratios **Increase:** PON1 activity
Pourteymour Fard Tabrizi et al English (30)	2010	Tabriz	Double-blind randomized controlled clinical trial	64	1-omega -3 fatty acids 4 g/d 2-Placebo	8 weeks	**Reduce: **Glucose, Insulin, and Insulin resistance

LH= Luteinizing hormone

TG=Triglyceride

LDL= Low density lipoprotein

DHEAS=Dehydroepiandrosterone sulfate

T=Testosterone

BMI=Weight and Body mass index

FBS=Fasting blood sugar

TC=Total cholesterol

MDA=Malondialdehyde

PTH=Parathyroid hormone

HDL=High density lipoprotein

PON1=Paraoxonase 1

HOMA-IR=Homeostatic model assessment- Insulin resistance index

## Discussion

In this study, 11 clinical trials were assessed that in 10 of them randomization was emphasized (25-34). One of these studies was quasi randomized trial (24). Three studies were about Calcium-vitamin D or calcitriol; 3 studies were about ω-3 fatty acids and 2 studies about N-acetyl cysteine; and 1 study about folic acid; 1 study about Zinc; and 1 study about Soy (24-34). 

**Calcium and vitamin D**

Some studies support the effect of vitamin D deficiency on pathophysiology of PCOS and even insulin resistance (36-38). Pal *et al *found that 3 months supplementation with vitamin D and calcium (Ca) can reduce androgens. They believe that vitamin D and Ca have a direct effect on the ovarian and/or adrenal steroid genesis pathway (10). Firouzabadi *et al* also found calcium and vitamin D supplementation can make a positive effect on weight loss, follicle maturation, menstrual regularity, and improvement of hyperandrogenism, in infertile women with PCOS (39). According to Thys-Jacobs *et al *calcium hemostasis disturbance can cause follicle growth disorders (40). 

In this review, 2 studies about calcium and vitamin D also showed a significant effect of these supplements on follicle growth and response to main treatment (28, 32). One study on calcitrol also shows an increase in PTH, systolic BP, and ovulation (31). Rashidi *et al* suggest combination of calcium-vitamin D therapy increases therapeutic effects of metformin treatment of menstrual disorders and maturation of follicles than metformin alone (28). 

**ω-3 fatty acids**

ω-3 fatty acids, at first was found in fatty fish. Fish oil that is the main source of dietary ω-3 fatty acids; have several healthy effects including anti-inflammatory, antithrombotic, antiarrhythmic and antiatherogenic effects (11). While insulin resistance is an important component in the pathogenesis of PCOS and on the other hand, this syndrome is associated with inflammatory factors, so polyunsaturated fatty acids (PUFA) may treat PCOS with the help to decrease insulin resistance and androgen excess (41, 42). Oner and muderris showed ω-3 also may be effective in decreasing hirsutism, BMI, LH, testosterone, insulin, Homeostatic model assessment (HOMA) levels, and increasing Sex hormone-binding globulin (SHBG) and TNF-α in women with PCOS (12). 

In this review, 3 studies were about the effects of ω-3 on hormonal and metabolic aspects of PCOS in Iranian women (29, 33, 35). All of these studies were RCTs with placebo group. Rafraf *et al* in their study found ω-3 fatty acids for 8 weeks can decrease total cholesterol (TC), triglyceride, low density lipoprotein (LDL), malonodialdehyde (MDA), and increase high density lipoprotein (HDL), but make no changes in total antioxidant capacity (TAC). These researchers concluded that ω-3 fatty acids are useful for PCOS women in order to reduce lipids and lipid peroxidation levels (34). Mohammadi *et al *also found 8 weeks supplementation with ω-3 fatty acids can reduce TC/HDL-C and LDL-C/HDL-C ratios and increase paraoxonase-1(PON1) activity in comparison with placebo. They believe using the ω-3 fatty acids is an appropriate approach to decrease cardiometabolic risks (29). Besides, Rafraf *et al* in another paper reported ω-3 fatty acid supplementation could make lower levels of glucose, insulin and insulin resistance but no significant changes in serum levels of high sensitive C-reactive protein in PCOS women. They concluded that ω-3 fatty acid supplementation is a helpful approach for PCOS metabolic disturbances although this need more studies (33).

**N-Acetyl cysteine (NAC)**

NAC (N-acetyl-cysteine) is an antioxidant that derivative from the amino acid L-cysteine. NAC can have effects on insulin receptor activity as well as insulin secretion and subsequently increase glucose utility (13). Besides, NAC has antiapoptotic activity and decreasing effects on homocystein (Hcy) levels. Diet cannot provide NAC, but its nutritional supplement is available (43). Fulghesu *et al* study showed NAC can have effect on levels of circulated insulin and insulin sensitivity in PCOS women with hyperinsulinemia (44). NAC is an appropriate choice for induction ovulation or augmentation in PCOS women and can be used as an adjuvant to Clomiphene Citrate (45). 

Gayatri *et al* showed significant effects of NAC on the clinical features, biochemical markers of insulin resistance, hormonal levels, anovulation, and oxidative stress inhibition in PCOS women. They recommended insulin reducing medications can be replaced by NAC because of its limited adverse effects (13). In this review, the study of Salehpour *et al* through a prospective double-blind clinical trial on 46 PCOS women showed 6 weeks use of NAC can increase ovulation rate and HDL levels and decrease weight, body mass index (BMI), and waist/hip ratio, fast blood sugar (FBS), serum insulin, total cholesterol, LDL levels, and HOMA-IR index while luteinizing hormone (LH), Follicle-stimulating hormone (FSH), prolactin, LH/FSH levels and glucose/insulin ratio were the same with no significant changes (27). Another study of Salehpour *et al* showed that using of NAC as an adjuvant in Clomiphene citrate cause an increase in number of follicles >18 mm, mean endometrial thickness on the day of hCG administration, ovulation and pregnancy rates with no adverse side-effects and no cases of ovarian hyperstimulation syndrome (26).

**Soy**

Soybeans are one of the best sources of proteins regarding the quality (contains most of the essential amino acids) and quantity (36-56% protein). Soy protein also contains fatty acids, saponins, isoflavones and phospholipids (46). There are many evidences that use of soy protein provides many desirable effects on weight and lipid metabolism (47). Soy also has antioxidant effect that may reduce oxidative lipid damage and subsequently, protect against cancer and cardiovascular diseases (48). Romualdi *et al* in a study on 12 obese, hyperinsulinemic, and dyslipidemia women with PCOS showed phytoestrogens supplementation improved total cholesterol, low-density lipoprotein and LDL/HDL ratio whereas had no effect on anthropometric features, the hormonal status, glucose and insulin metabolism and menstrual cycles (49). 

Khani *et al* in their study about soy phytoestrogen (Genistein) effects on PCOS women found although HDL and FSH serum levels in Genistein and placebo group before and after treatment didn’t show any significant differences; but LH, TG, LDL, DHEAS, and testosterone levels were significantly decreased in case group. These researchers concluded that Genistein can be useful for cardiovascular and metabolic disorders prevention in PCOS women (24).

**Folic acid**

Polycystic ovary syndrome is one of the conditions that are associated with elevated homocysteine levels. Homocysteine is a product of methionine metabolism and can cause cytotoxic effects on vascular endothelium. So hyperhomocysteinemia is a risk factor for atherosclerosis, thromboembolism, hyperinsulinemia, and consequently cardiovascular disease (50). Folic acid is one of the supplements which have a well-known physiological effect on Hcy reduction. So it can improve endothelial function either due to its effect on Hcy or even via other mechanisms that are not associated to Hcy (51, 52). According to Palomba *et al*, 6 months treatment with metformin and folic acid, could cause a significant improvement in all the markers of structure and function of the vascular endothelium and this improvement was significantly different between folic acid supplementation and placebo groups (53). 

Regarding this matter, Kazerooni *et al* in a prospective clinical trial on 70 Iranian hyperhomocysteinemic PCOS women reported that 3 months folic acid supplementation could significantly decreased Hcy levels in these women regardless of insulin resistance status although this reduction was higher in women without IR. These researchers believe that IR can have effect on Hcy responses to folic acid (25). 

**Zinc**

Biochemical role Zinc (Zn), one of the most important trace elements, is essential for more than 300 different cellular processes. Zn also is a basic element for many vital functions including homeostasis, immune responses, oxidative stress, and apoptosis and in other words, for health, either physically or mentally. Zinc also is involved in fertility and reproduction (54, 55). Besides, zinc is important for insulin synthesis and action in both, normal and diabetes mellitus condition (56). 

Although some studies didn’t find any significant differences between women with and without PCOS regarding to serum Zn levels, but Tabrizi *et al* in a randomized, double-blind, placebo-controlled parallel groups trial on 65 PCOS women showed that 8 wk supplementation with 50 mg of zinc in the zinc sulphate form can arise serum zinc significantly and also reduce homeostasis model of assessment-insulin resistance score, fasting serum total cholesterol, LDL-C, triglyceride, testosterone, and TG/HDL-C ratio in comparison with placebo but anthropometric indices and systolic-and diastolic blood pressure didn’t show any significant changes in both groups (57). They believe zinc supplementation for PCOS women has some beneficial effects on cardio metabolic risk factors (30).

## Conclusion

The results of all reviewed studies in this paper showed that antioxidants and vitamins have positive effects in management of PCOS and its' complications, although it seems more studies is necessary in this field because evidences are not enough to identify an optimum antioxidant management in women with PCOS. There is some strength in the present study which needs to be addressed. First, all studies were clinical control trials (first degree quality evidences) and sampling method in 10 from 11 of them was random. Therefore, results are more reliable. Secondly, all of reviewed studies were performed in Iran. This study has some limitations too. In some studies sample size was low and it may affect reliability of results. In addition we couldn’t include all of studies because all full texts were not accessible.

## Note

***This article extracted from Ph.D. thesis. (Leila Amini)***
